# Bilateral Neonatal Testicular Torsion; Hidden Surgical Nightmare

**DOI:** 10.3389/fped.2018.00318

**Published:** 2018-11-20

**Authors:** Tariq O. Abbas, Mansour Ali

**Affiliations:** ^1^Weill Cornell Medicine- Qatar, Ar-Rayyan, Qatar; ^2^Pediatric Surgery, Hamad Medical Corporation, Doha, Qatar; ^3^College of Medicine, Qatar University, Doha, Qatar

**Keywords:** neonatal, testicular torsion, perinatal, exploration, extravaginal

## Abstract

Perinatal testicular torsion is a relatively rare event that remains unidentified in many situations and managed only after an avoidable delay of time. Its current management approaches include watchful observation, delayed contralateral orchiopexy, and emergent contralateral orchiopexy. On the other hand, bilateral torsion is now being more frequently reported. However, the assessment of the contralateral testis through physical examination and imaging can be inaccurate in cases of perinatal torsion. We report a case of prenatal testicular torsion with incidentally discovered metachronous contralateral extravaginal testicular torsion. Therefore, immediate surgical intervention is recommended both when uni- or bilateral testicular torsion is suspected. Whenever possible, affected testes should be preserved as some endocrine function may be retained.

## Introduction

Neonatal testicular torsion (NTT) was first described by Taylor et al. in 1897 (1). It can be either unilateral or, less frequently, bilateral. In addition, bilateral NTT can be synchronous or metachronous. However, the exact time of the beginning of the torsion is difficult to determine. A high index of suspicion is mandatory for timely diagnosis of perinatal torsion even in the presence of “normal” other side. However, management plan and mode of intervention is highly controversial. We present here a case of bilateral NTT and challenge the importance of earlier surgical exploration whenever doubt exist. Moreover, recent insights into this condition were reviewed.

## Case report

**Figure 1:**
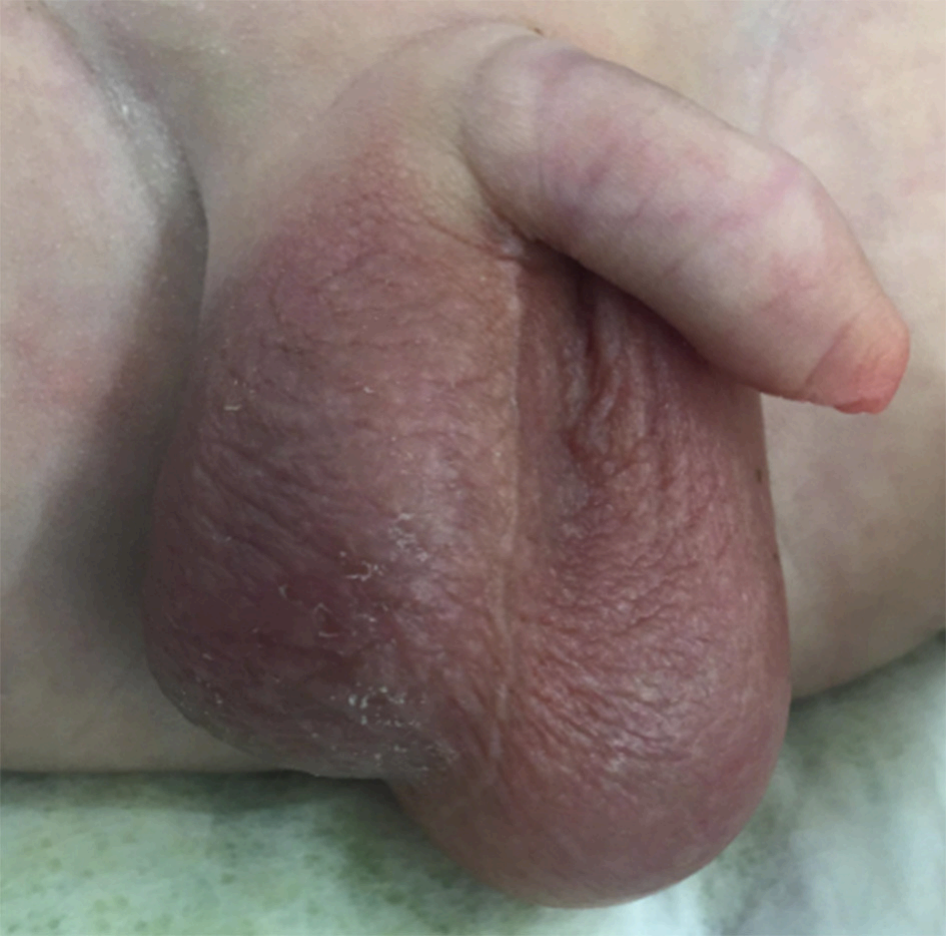
Preoperative photo of the external genitals.

**Figure 2:**
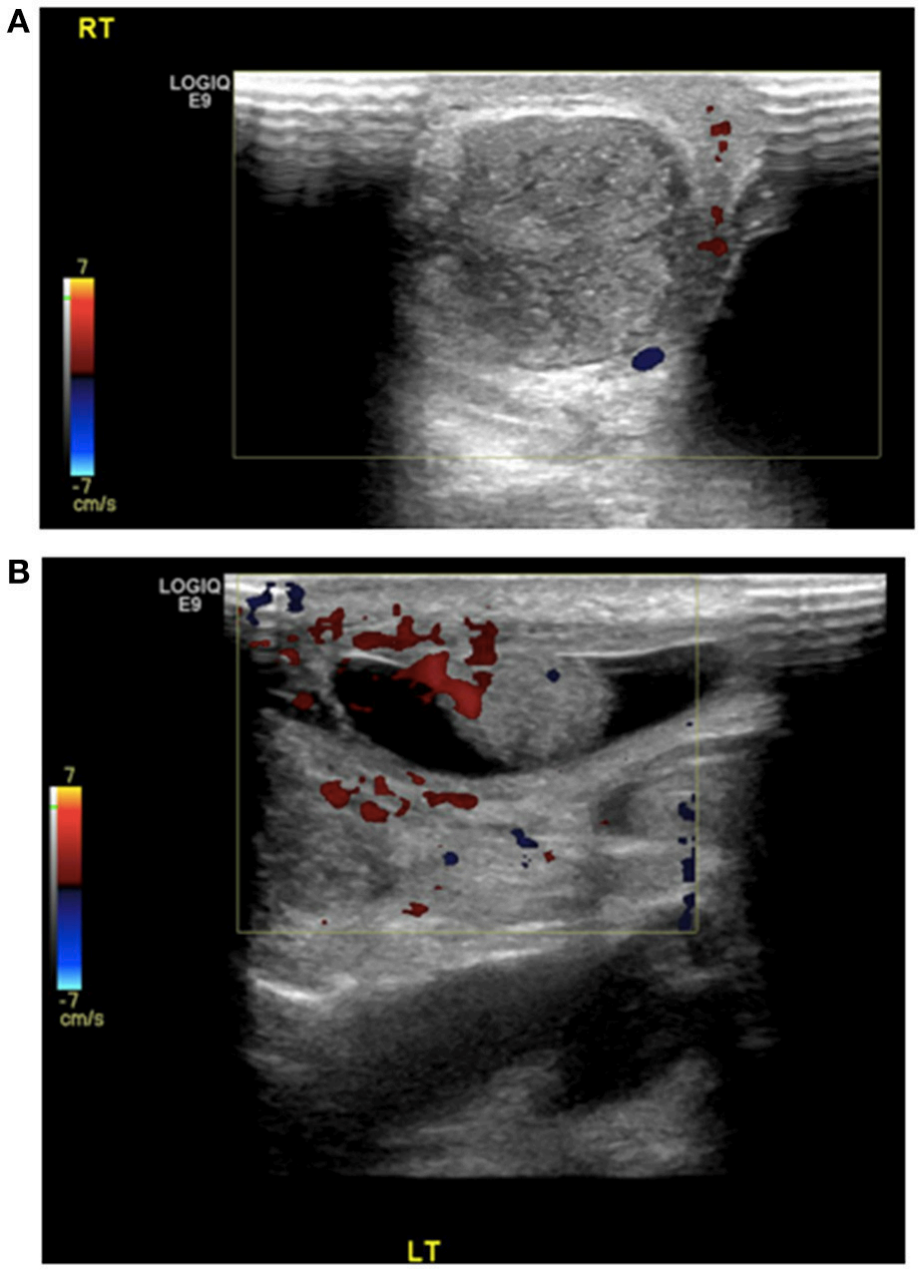
Doppler US showed **(A)** no blood flow to the right testis with **(B)** preservation of the vascularity of the left testis.

**Figure 3:**
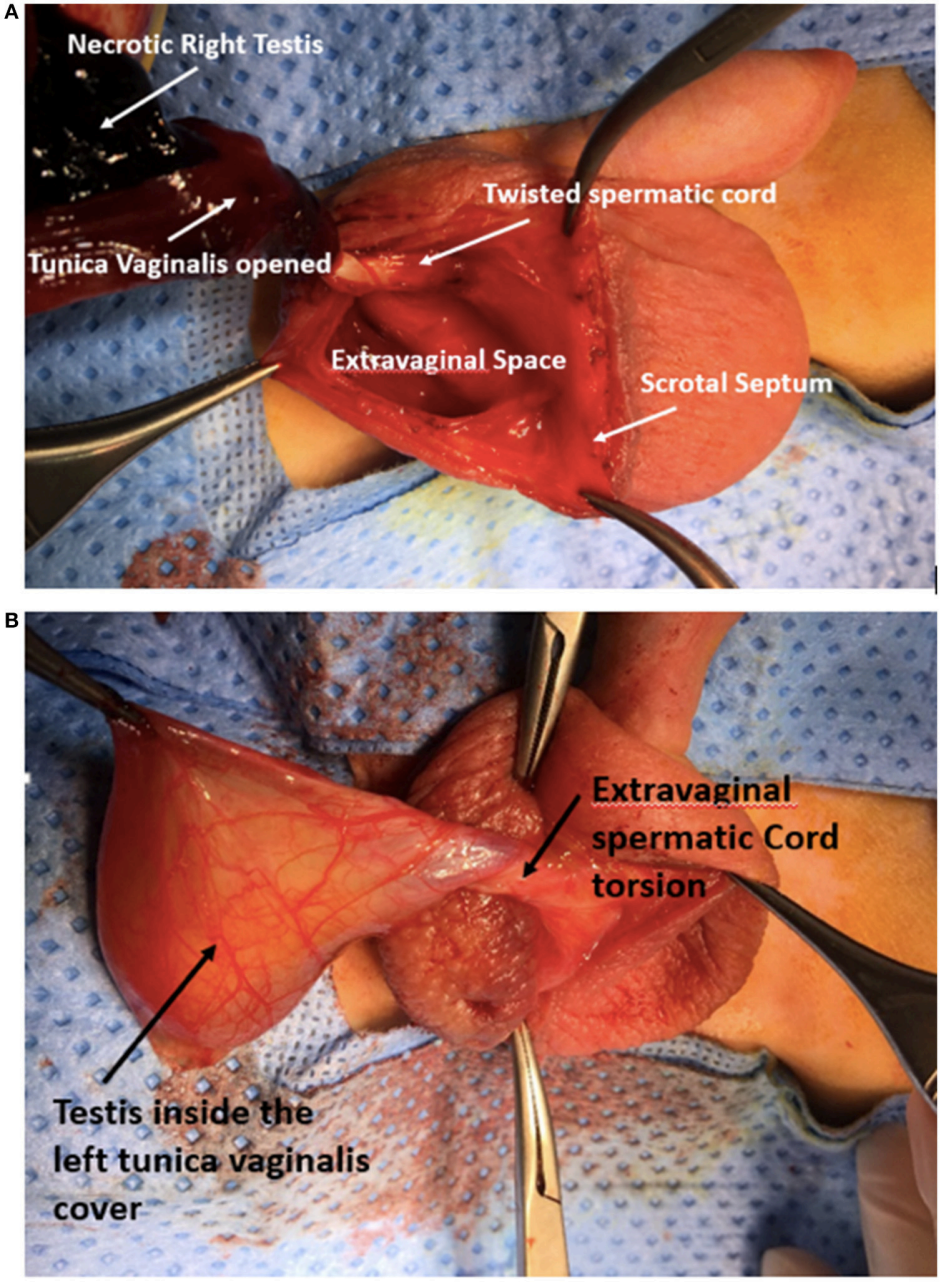
Intraoperative photo showing **(A)** extra-vaginal torsion of the right side with necrotic testis after opening the tunica vaginalis, **(B)** left side early onset of extra-vaginal TT and tunica vaginalis freely moving within the hemiscrotal sac with viable testis within it.

A 2-day old, male baby, product of a normal vaginal delivery, had a right hemi-scrotal swelling since birth, with bluish scrotal pigmentation (Figure 1). General examination was normal. Color Doppler Ultrasound of the scrotum, showed absence of vascularity in the right testis and maintained blood flow to the other one (Figure 2). Right scrotal exploration was done through midline raphe incision and revealed right extra-vaginal testicular torsion with a necrotic testis (Figure 3A). Contralateral exploration showed extra-vaginal testicular torsion with normal vascularity (Figure 3B).

Right orchiectomy with 3-point fixation of the left testis was performed. Histological examination confirmed right testicular coagulative necrosis. Follow-up at 3 months showed that the left testis was normal in size with good blood flow by Doppler signal.

## Discussion

Neonatal scrotal swelling has several causes, including: birth trauma, epididymitis, appendicitis, hydrocele, and Testicular Torsion. Although neonatal testicular torsion is considered a rare clinical entity, several reports indicate that it is recently more frequently encountered. Most of the cases happen before birth and only about 20% occur following delivery (2, 3) About 150 perinatal TT have been reported since 2010, with a significant percentage of bilateral cases (4). However, bilateral NTT is much rarer and was not described until 1967 (5). It is synchronous in 50–80% of the cases (6–9).

Neonatal testicular torsion is of the extravaginal type where all scrotal contents twist around the spermatic cord (10). Genetic predisposition might play a rule in the etiology of NTT as several familial cases has been reported (11, 12). Furthermore, *Insl3* knockout mice was shown to have both intraabdominal undescended tests and spontaneous TT. As a result, the INSL3 hormone and its receptor, RXLF2, have been pointed to be candidate genes for testicular torsion (13, 14). This explains the occurrence of prenatal TT and vanished testes events in humans. However, human post-natal TT takes place in the scrotal sac and not inside the abdomin, as in the mice. However, Wang et al. studied DNA samples of 39 males (11 neonatal: 21 peri-pubertal; 7 post-pubertal) with testicular torsion. Bilateral testicular torsion was present in 2/11 (18%) neonatal and 2/28 (7%) older cases. A positive family history of TT was present in about one third of both groups. He did not detect any functionally significant mutations in INSL3 or RXFP2, questioning that mutations in these genes are not a common *de novo* or inherited cause for testicular torsion (15).

Clinical diagnosis of perinatal TT can be a challenging task as most of the cases are asymptomatic, with no tenderness but slight scrotal induration and discoloration. Although Doppler US is frequently utilized as an adjunct to ease that diagnosis of borderline cases, it has its own limitations (16–18). On the other hand parenchymal heterogeneity of the testes was shown to correlate significantly with testicular unsalvagability (19). Therefore, some people suggest that in the presence of parenchymal heterogeneity, orchidectomy, and contralateral fixation can be performed in a less urgent manner (20). However, we do not recommend this approach as there is still good chance of salvagability of testes (21); moreover, there is a real danger of contralateral synchronous TT as we had in this case. On the other hand, Martin et al. has suggested that routine contralateral orchiopexy is not needful in cases of NTT as the presence of bell-clapper anomaly in the other “normal” testis is very rare (22). However, this is arguable by the fact that contralateral testicular torsion in cases of perinetal TT is from the extra-vaginal variety, therefore we still propose contralateral fixation.

Lastly, prompt intervention could save the patients the pain and potential complications that they might have. Recently, near-infrared spectroscopy (NIRS) was shown to provide noninvasive transcutaneous monitoring of deep tissue oxygen saturation (%StO2). It was demonstrated that %StO2 values in the affected testis were significantly lower than unaffected testis (23). Nevertheless, no single investigation is flawless and, when it comes to TT, it is always advised to rely on the detailed history and a thorough physical exam to reach the diagnosis and surgical intervention should take place whenever the diagnosis is in doubt.

Perinatal torsion has been subdivided into prenatal and postnatal (event occurring from birth to 1 month of life) torsion. Management of prenatal neonatal testicular torsion has a lack of consensus in terms of surgical timing and need for contralateral fixation (24). Likewise, there is a scarcity of reports on testicular salvagability in this group of patients secondary to the established infarction (25–27). Moreover, relieving the “testicular compartment” (28) might not be beneficial, but worth trying in selective cases. Metachronous contralateral PTT has been reported and emergent exploration with contralateral orchiopexy was raised as the best method of intervention (29). It has recently been shown that 25 orchiopexies are needed to potentially prevent the loss of a contralateral TT (30). On the other hand, management of synchronous bilateral PTT appears has more consensus, and immediate exploration and orchiopexy rather than orchiectomy is most frequently advocated as Leydig cells tolerate severe ischemia more than other cell types of the testes maintaining the capacity to endogenously produce testosterone. Therefore, the capacity of endogenous testosterone production may continue (4, 31). However, leaving nechrotic testes behind carries the risk of abscess formation and wound infection. On the other hand, postnatal TT should be managed with immediate surgical exploration similar to that an older patients with TT as there is a real potential for testicular conservation (32, 33).

In conclusion, immediate surgical intervention is recommended in suspected bilateral NTT as well as in cases of unilateral torsions with “normal” contralateral one. Whenever possible, affected testes should be retained as endocrine function has the potential to persist.

## Ethics statement

The project has been approved by the Medical Research Centre (MRC) of Hamad Medical Corporation (HMC). Study Number (MRC-4-18-230).

## Author contributions

TA prepared and wrote the article. MA has reviewed and critically analyzed the manuscript.

## Photography consent

Written informed consent was obtained from the parent of the patient for the publication of this case report.

### Conflict of interest statement

The authors declare that the research was conducted in the absence of any commercial or financial relationships that could be construed as a potential conflict of interest.
